# Evaluation of the cardiorespiratory and antinociceptive effects of the total intravenous anesthesia technique with xylazine-butorphanol-propofol compared to isoflurane in calves under experimental laparotomy

**DOI:** 10.3389/fvets.2025.1688448

**Published:** 2025-10-17

**Authors:** Shogo Sato, Chihiro Kanno, Akihiro Matsuura, Yosuke Maeda, Fumiaki Takahashi

**Affiliations:** ^1^Laboratory of Clinical Veterinary Medicine for Large Animals, School of Veterinary Medicine, Kitasato University, Towada, Aomori, Japan; ^2^Laboratory of Animal Behavior, School of Veterinary Medicine, Kitasato University, Towada, Aomori, Japan

**Keywords:** cattle, clinical safety, general anesthesia, heart rate variability, isoflurane, surgical stress, xylazine-butorphanol-propofol combination

## Abstract

A novel total intravenous anesthesia (TIVA) technique combining xylazine (0.1 mg/mL), butorphanol (0.01 mg/mL), and propofol (2 mg/mL) in a 5% dextrose solution (XBP) has shown promising results in calves. In the present study, we compared XBP-TIVA with isoflurane (ISO) inhalation anesthesia during experimental laparotomy in calves, evaluating cardiorespiratory and antinociceptive effects. Fourteen clinically healthy male and female calves (body weight: 28.5–155.0 kg; age: 17–186 days) were randomly assigned to receive XBP-TIVA (continuous infusion at a rate of 6 mL/kg/h) or ISO inhalation anesthesia (end-tidal concentration: 1.3 ± 0.1%) for 60 min during laparotomy. We measured rectal temperature (RT), heart rate (HR), arterial blood pressure, and arterial blood gas values. Then, HR variability (HRV) components and stress hormones were measured to analyze autonomic nervous system and neuroendocrine responses to nociceptive stimuli. Measurements were taken before drug administration (baseline) and at 5-min or 15-min intervals after anesthesia maintenance. Nociceptive stimuli were administered, including skin and muscle incision, intestinal manipulation, muscle suture, and skin suture. The statistical significance level was set at *p* < 0.05. The XBP group maintained significantly higher RT throughout anesthesia. HR decreased significantly from baseline in both groups. Mean arterial pressure remained significantly higher in the XBP group. The XBP group demonstrated significantly lower arterial partial pressure of carbon dioxide values than the ISO group throughout anesthesia maintenance. During intestinal manipulation, the normalized high-frequency component significantly decreased while the low-frequency/high-frequency ratio significantly increased. Norepinephrine concentrations in both groups demonstrated a significant decrease from baseline values prior to the nociceptive stimuli; however, the difference disappeared during and before the stimulus. Epinephrine concentrations were lower than baseline values at all measurement points in both groups; however, a significant increase from before to after the nociceptive stimulus was observed only in the ISO group. In conclusion, compared to ISO anesthesia, XBP-TIVA maintained superior cardiorespiratory stability, higher RT, and more efficient ventilation. XBP demonstrated superior nociceptive suppression, though both protocols suggested sympathetic responses to visceral pain. These findings suggest that XBP-TIVA may be a safe, effective, and potentially superior alternative to isoflurane inhalation anesthesia for surgical procedures in calves.

## 1 Introduction

Pain management in farm animals is ethically important, and highly invasive surgeries should be performed under general anesthesia whenever possible (1). In field conditions, surgical procedures are often performed under sedation and local anesthesia (2, 3). However, this approach frequently provides inadequate pain relief and muscle relaxation. This can potentially cause patient movement during surgery, which may affect procedural success rates. Maintaining an anesthetized state often requires the use of additional sedatives and analgesics, rescue dosing. This increased rescue dosing tends to prolong surgery (4). An extended surgical duration places a significant burden on both patients and surgeons, making a stable and sufficient anesthetic depth essential for ensuring surgical safety. Abdominal surgeries (5) and fracture repairs (6), which cause significant trauma and require extended time in calves are commonly performed under general anesthesia. However, general anesthesia is difficult to implement in field environments due to equipment and personnel limitations (1). Inhalation anesthesia, requiring specialized equipment, presents even greater implementation challenges. In contrast, total intravenous anesthesia (TIVA) can be easily used in the field conditions without specialized equipment (3).

We have previously reported on a novel TIVA protocol for calves (XBP), which combines xylazine (0.1 mg/mL), butorphanol (0.01 mg/mL), and propofol (2 mg/mL) in a 5% glucose solution (1). Although XBP may be useful for general anesthesia in calves, there is insufficient knowledge about its stability and safety for open abdominal surgery.

The autonomic nervous system (ANS) reflexively responds within seconds to stress signals through both the parasympathetic nervous system (PNS) and sympathetic nervous system (SNS) pathways (7). Surgical stress triggers SNS responses, resulting in the release of catecholamines from the adrenal medulla and cortisol (CSL) from the adrenal cortex, which affect the immune system and hemodynamics (8, 9). The heart rate variability (HRV) is a non-invasive method for evaluating ANS activity by measuring variations in the temporal distance between consecutive heartbeats (R-R intervals) (10). A frequency domain analysis classifies R-R intervals into very-low-frequency (VLF), low-frequency (LF), and high-frequency (HF) components. Furthermore, normalized low-frequency (LF (n.u.)) and high-frequency (HF (n.u.)) represent the relative values of each power component to total power, excluding VLF. In cattle, HRV has been reported in studies evaluating behavioral, psychological, and physiological stress (11–14), puerperal fever (15), and pain associated with surgical castration (16). Therefore, the combination of HRV analysis and stress hormone measurements may provide a more accurate assessment of autonomic nervous system balance and nociceptive stress during laparotomy under general anesthesia for cattle.

In the present study, we evaluated the cardiorespiratory function, anesthetic depth, quality of recovery, autonomic function as assessed by HRV analysis, and antinociceptive effects measured through hormone levels when using ISO inhalation anesthesia versus XBP-TIVA for experimental laparotomy in calves. We hypothesized that this systematic assessment would establish a comprehensive safety profile for XBP-TIVA, enhancing its potential for implementation as an innovative anesthetic technique in clinical veterinary practice for cattle.

## 2 Materials and methods

### 2.1 Animals

In the present study, we used 14 calves with a median [minimum-maximum] body weight of 53.5 [28.5–155.0] kg and age of 62.0 [17.0–186.0] days (Table 1). All calves underwent a physical examination and a complete blood count test. Although some calves presented with localized abnormalities, the results of their physical examinations were deemed normal. Therefore, based on the American Society of Anesthesiologists (ASA) grade of physical status (17). All calves classified as ASA grade I or grade II were included in the present study.

**Table 1:** Profile of the animals used in this study.

No.	Breed	Age (days)	Sex	Body weight (kg)	Animal condition	ASA grade
XBP group						
Calf 1	Japanese Black	116	Female	75.5	Lameness (Systemically healthy)	II
Calf 2	Holstein	186	Steer	155.0	Healthy	I
Calf 3	Holstein × Japanese Black crossbred	128	Male	81.5	Joint swelling (Systemically healthy)	II
Calf 4	Holstein	27	Male	28.5	Healthy	I
Calf 5	Holstein	17	Male	38.0	Healthy	I
Calf 6	Holstein	19	Female	36.0	Healthy	I
Calf 7	Japanese Black	62	Male	62.0	Umbilical hernia (Systemically healthy)	II
ISO group						
Calf 8	Holstein × Japanese Black crossbred	50	Female	48.5	Healthy	I
Calf 9	Holstein × Japanese Black crossbred	76	Male	58.0	Healthy	I
Calf 10	Holstein × Japanese Black crossbred	60	Male	50.0	Healthy	I
Calf 11	Holstein × Japanese Black crossbred	62	Female	57.0	Healthy	I
Calf 12	Japanese Black × Holstein crossbred	51	Female	50.0	Healthy	I
Calf 13	Holstein	62	Male	46.5	Healthy	I
Calf 14	Holstein	186	Male	150.5	Nystagmus (Systemically healthy)	II

XBP total intravenous anesthesia via continuous infusion of xylazine, butorphanol, and propofol, ISO inhalation anesthesia with isoflurane, ASA the American Society of Anesthesiologists grade of physical status.

### 2.2 Study design

The present study was conducted using a prospective, permuted block randomization, open-label clinical trial design. Calves (*n* = 14) were divided into two groups: the XBP group (*n* = 7) and ISO group (*n* = 7). The XBP group received a continuous rate infusion (CRI) of a combination of xylazine (10 μg/kg/min; Celactal 2%, Elanco Japan, Tokyo, Japan), butorphanol (1 μg/kg/min; Vetorphale® 5 mg, Meiji Animal Health Co., Kumamoto, Japan), and propofol (200 μg/kg/min; Propofol 1%, Maruishi Pharmaceutical, Osaka, Japan) mixed in 5% dextrose solution (Otsuka Dextrose Injection 5% 250 mL and 500 mL, Otsuka Pharmaceutical Factory, Inc., Tokushima, Japan) at a rate of 6 mL/kg/h throughout the maintenance period. No loading bolus was administered before initiating the CRI in the XBP protocol. The ISO group received ISO (Isoflurane for animals, Mylan N. V., Tokyo, Japan) with the vaporizer dial set between 1.3–3% to maintain an end-tidal ISO concentration (EtIso) of 1.3 ± 0.1%, along with a concurrent intravenous infusion of 5% dextrose solution (6 mL/kg/h). In the present study, we evaluated cardiorespiratory function, anesthetic depth, recovery quality, autonomic function through HRV analysis, and surgical stress response via hematological and hormonal parameters. The measurement of relevant parameters occurred at the baseline (BL), prior to the administration of the pharmaceutical agent under investigation, as well as at regular intervals of 5 and 15 min thereafter. Throughout the present study, anesthetic management was performed by the same researcher. Sample collection was performed by veterinary students under the instruction and supervision of the same researcher. Samples with hemolysis and data with poor recordings were excluded from the analysis.

### 2.3 Vascular access procedure

On the day before the anesthesia experiment, an arterial line (the left carotid artery) and venous line (the left jugular vein) were placed in all calves, as described in previous studies (1, 18). Calves were fasted for approximately 18 h prior to anesthesia to prevent bloat and reflux of ruminal contents. They had free access to water. Following the completion of the procedure, feed was provided.

Xylazine hydrochloride (0.2 mg/kg) was administered intravenously to both groups, and calves were placed in the right lateral recumbent position on a mat. Propofol (2 mg/kg) was injected intravenously, followed by tracheal intubation and connection to a compact anesthesia apparatus (COMPACT - 15, Kimura Medical Instrument Co., Ltd., Tokyo, Japan). All calves inhaled ISO (anesthetic vaporizer dial: 1.3–3%) and pure oxygen (5 L/min) in a semi-closed respiratory circuit and maintained spontaneous respiration throughout the procedure. The left cervical groove surrounding the site was extensively shaved, and the surgical site was cleansed with 7.5% povidone-iodine solution (Isodine® Scrub Solution 7.5%, Mundipharma K. K., Tokyo, Japan), followed by 2% povidone-iodine solution (Isodine® Solution for Veterinary Use, Mundipharma K. K., Tokyo, Japan) and 70% isopropanol (70% Isopropanol for Disinfection, Nikko Pharmaceutical Co., Ltd., Gifu, Japan). Regarding infiltration anesthesia, procaine hydrochloride (10 mL/body; Procaine hydrochloride injection for animals, Kyoritsu Seiyaku Corporation, Tokyo, Japan) was injected subcutaneously at the surgical site.

A 10- to 15-cm skin incision was made cephalocaudally along the slightly dorsal aspect of the cervical groove with a No. 23 scalpel blade (Replacement Blade No. 23; Feather Safety Razor Co., Ltd., Osaka, Japan). The fascia of the brachiocephalicus muscle was sharply incised, and the muscle fibers were bluntly divided by hand. The longus capitis and sternomastoideus muscles were bluntly dissected to expose the carotid sheath. Forceps were used to bluntly open the carotid sheath and separate the left carotid artery and vagus nerve trunk. The brachiocephalicus and longus capitis muscles were sutured with absorbable threads (Synthesorb USP 3 + 4, Kawasaki Biological Science Research Institute Co., Ltd., Tokyo, Japan). The left carotid artery was then placed subcutaneously. An 18-gauge, 32-mm catheter (Surflo IV Catheter, Terumo Corporation, Tokyo, Japan) was implanted in the left carotid artery using a soft tissue adhesive (Aron alpha A “Sankyo,” Daiichi Sankyo Company, Limited, Tokyo, Japan). The 18G catheter was connected to the blood pressure tube of a pressure transducer (Meritrans DTXPlus Disposable Transducers DT4812, Merit Medical Japan K. K., Tokyo, Japan) and a three-way stopcock (Terumo Corporation, Tokyo, Japan) (A-line). The interior of the catheter was filled with heparinized saline (20 IU/mL) prepared by mixing heparin sodium (Heparin Sodium Injection 10,000 units/10 mL, Nipro Corporation, Osaka, Japan) and normal saline (Otsuka Normal Saline 500 mL, Otsuka Pharmaceutical Factory, Inc., Tokushima, Japan). Benzyl penicillin procaine (1.5 million IU/mL; Suspended Aqueous Procaine Penicillin G Injection NZ, Nippon Zenyaku Kogyo Co., Ltd., Fukushima, Japan) was applied to the surgical site. An 18-gauge catheter was placed subcutaneously, and the skin was sutured with non-absorbable threads (Suprylon USP 3 + 4, Kawasaki Biological Science Research Institute Co., Ltd., Tokyo, Japan) for continuous interlocking sutures. A 16-gauge, 64-mm catheter (Surflo IV Catheter, Terumo Corporation, Tokyo, Japan) was placed in the left jugular vein and connected to an extension tube (Extension Tube, JMS Co., Ltd., Hiroshima, Japan) and a three-way stopcock (V-line). The V-line was filled with heparinized saline (20 IU/mL) (V-line).

The A- and V-lines were fixed to the neck with an adhesive bandage (New-HALE AKT, New Halex Co., Iwata, Shizuoka, Japan). Benzyl penicillin procaine (1.5 million IU/mL) was injected intramuscularly for infection prevention, and tranexamic acid (Tramlin Injection, 20–50 mL/body, Nippon Zenyaku Kogyo Co., Ltd., Fukushima, Japan) was injected intravenously for hemostasis. After the termination of ISO anesthesia and confirmation of the swallowing reflex, atipamezole (20 μg/kg; Antisedan®, Nippon Zenyaku Kogyo Co., Ltd., Fukushima, Japan) was intravenously injected. Calves were then allowed to recover from anesthesia. The calves were promptly fed after confirming their complete recovery from anesthesia. At 16:00 on the same day and at 9:00 the next day, the interiors of the A-line and V-line catheters were flushed with heparinized saline (20 IU/mL). In two calves in the XBP group, an A-line was created in the right carotid artery because the left A-line was disabled before the experiment began.

### 2.4 Anesthesia experiment protocol

The anesthesia experiment started at 14:00 the day after the vascular access procedure. Calves were fasted for approximately 22 h prior to the start of this experiment and had free access to water (1). The amount of drug used in the anesthesia experiment was calculated from body weight measured the day before or the day of the experiment. To simplify dose calculations, the body weights of some animals were either rounded to the nearest 10 or truncated after the decimal point.

Calves were allowed to rest in the standing position for at least 10 min. In both groups, xylazine hydrochloride (0.2 mg/kg) was administered intravenously, and calves were placed in the supine position on the operating table. Subsequently, propofol (2 mg/kg) was injected intravenously (1), followed by tracheal intubation and connection to the respiratory circuit of the anesthesia machine (Acoma Anespirator KMA-1300Vi or Acoma Animal Anesthesia Machine NS-7000A, Acoma Medical Industries Co., Ltd., Tokyo, Japan). Subsequently, the respective anesthetic maintenance protocols were initiated in XBP and ISO groups. During the maintenance phase of anesthesia, both groups received oxygen at a rate of 5 L/min through a semi-closed breathing circuit. Pressure-controlled mechanical ventilation was implemented as well. The inspiratory pressure and the ventilation rate for respiratory management were adjusted as needed to maintain a respiratory rate of 8–12 breaths/min. In addition, end-tidal carbon dioxide pressure (EtCO_2_) was meticulously maintained within the range of 35–40 mmHg. The measurement was obtained by collecting expired air connected between the breathing circuit and tracheal tube using a capnometer on the vital signs monitor (Bioscope AM130, Fukuda M. E. Kogyo Co., Ltd., Tokyo, Japan).

Sixty minutes after the start of anesthetic maintenance, anesthetic administration was stopped and assisted breathing was provided by manual positive pressure ventilation until spontaneous breathing occurred. Once spontaneous breathing was confirmed, oxygen inhalation was stopped, and calves were transferred from the operating table to a mat. After the swallowing reflex was confirmed, the tracheal tube was removed. Atipamezole (20 μg/kg) was then administered intravenously.

### 2.5 Blood sampling

Blood samples were collected from the A-line at five time points: BL and 15 (T15), 30 (T30, before skin incisions), 45 (T45), and 60 (T60) minutes after the initiation of anesthesia maintenance. To prevent sample dilution and the contamination of samples, 3–5 mL of blood was initially withdrawn using a 20-mL syringe (Terumo syringe 20 mL, Terumo Corporation, Tokyo, Japan) and discarded before collecting the samples. Collected blood was distributed into heparin sodium-rinsed 2.5-mL syringes (Terumo syringe 2.5 mL, Terumo Corporation, Tokyo, Japan), heparin sodium-containing tubes (Venoject II Vacuum Blood Collection Tube, Terumo Corporation, Tokyo, Japan), and ethylenediaminetetraacetic acid (EDTA)-containing tubes (Venoject II Vacuum Blood Collection Tube, Terumo Corporation, Tokyo, Japan). After blood collection, the catheter was flushed with 10–15 mL of heparinized saline (20 IU/mL). Blood in heparin sodium-rinsed syringes was immediately used for a blood gas analysis and blood glucose level (BGL) measurements. Blood in EDTA-containing tubes was used immediately for hemoglobin (Hb). After the experiment, the blood in heparin sodium-containing tubes was centrifuged at 1,580 × *g* for 15 min, and the plasma obtained was stored at −80 °C.

### 2.6 Laparotomy protocols

The left paramedian area of calves was shaved extensively, and the surgical site was cleansed with 7.5% povidone-iodine solution, followed by 2% povidone-iodine solution and 70% isopropanol. A 15-cm proposed incision line was marked with a surgical marker (Skin Marker, Koken Co., Ltd., Tokyo, Japan). Procaine hydrochloride (10 mL/body) was administered subcutaneously along the proposed incision line for local infiltration anesthesia. Between 30 and 35 min (T30-T35) after the initiation of anesthesia maintenance, incisions were made along the proposed incision line through the skin, muscle layer, and peritoneum in sequence. Manual intestinal manipulation was then performed between 35 and 40 min (T35-T40). Between 40 and 50 min (T40-T50), the peritoneum and muscle layer were closed using continuous sutures with absorbable threads (USP 3 + 4), followed by skin closure with continuous interlocking sutures with non-absorbable threads (USP 3 + 4) between 50 and 55 min (T50-T55). All surgical procedures were performed by the same surgeon. The evaluation of pain in the calves during surgery was conducted using a three-level pain scale that has been previously reported (2) with slight modification: Grade 0: no movement; Grade I: slight limb movements, muscle contractions at the surgical site, marked increase in respiratory rate; and Grade II: evident defensive reactions. We set Grade II as the criterion for experiment termination. If such behavior was observed, we would switch the XBP group to ISO inhalation anesthesia or increase the ISO concentration in the ISO group, then immediately close the abdominal cavity and terminate the experiment.

### 2.7 Assessment of cardiorespiratory function

Cardiorespiratory parameters were measured at BL and at 5-min intervals from 10 to 60 min after the start of anesthesia maintenance (T10, T15, T20, T25, T30, T35, T40, T45, T50, T55, and T60). Rectal temperature (RT) was measured using a mercury thermometer. HR was recorded with a Polar H10 transmitter (Polar Electro Oy, Kempele, Finland), which was connected to electrode belts that were attached to the shaved area around the chest and monitored successively via a Polar watch (Polar Pacer, Polar Electro Oy, Kempele, Finland). The Polar H10 transmitter and electrode belts were attached to the thoracic circumference on the morning of the experiment. Before attaching the belt, the thoracic area was cleaned with 70% isopropanol, and ultrasound gel was applied to the electrode portions of the belt to ensure proper contact with the body. The belt was further fixed with adhesive bandages. Mean arterial pressure (MAP), systolic arterial pressure (SAP), and diastolic arterial pressure (DAP) were measured using vital signs monitor connected to a pressure transducer positioned at the level of the right atrium and attached to an arterial line. Due to the unavailability of precise BL values in one of the subjects in the ISO group, an A-line was inserted into the right carotid artery 11 days after the anesthesia experiment. The following day, BL-MAP values were re-examined and used in the analysis.

A blood gas analysis was performed on arterial blood samples collected at BL and at 15-min intervals (T15, T30, T45, and T60). A blood gas analyzer (Gastad navi, Techno Medica Co., Ltd., Yokohama, Japan) was used to measure arterial blood pH (pHa), arterial carbon dioxide partial pressure (PaCO_2_), and arterial oxygen saturation (SaO_2_), with values corrected for the concentration of Hb and RT. EtCO_2_ was measured at 15-min intervals (T15, T30, T45, and T60) by collecting the expired air connected between the breathing circuit and tracheal tube using a capnometer on the vital signs monitor.

### 2.8 Assessment of depth of anesthesia and quality of recovery

The depth of anesthesia was evaluated based on the presence or absence of the palpebral (touching the eyelid) and corneal (touching the cornea) reflexes at BL and 15-min intervals (T15, T30, T45, and T60). The quality of recovery from anesthesia was evaluated by measuring the time required for the swallowing reflex to return after discontinuing anesthetic administration.

### 2.9 HRV analysis

HR data recorded by the Polar watch were transferred to Polar Flow software (ver. 3.5.0, Polar Electro Oy, Kempele, Finland) to obtain R-R interval data. A frequency domain analysis was performed using an autoregressive model with the Kubios HRV Premium HRV analysis tool (ver. 3.5.0, Kubios Oy, Kuopio, Finland) (19). HRV frequency bands were set to the LF (0.04–0.10 Hz) and HF (0.10–0.80 Hz) components (14). In addition to LF and HF power, data recording included normalized LF (LF (n.u.) = LF/ (Total power−VLF) × 100), normalized HF (HF (n.u.) = HF/ (Total power−VLF) × 100), and LF/HF ratio (LF/HF), calculated according to a previous study (10). The HRV analysis was performed on 5-min HR data recordings obtained during the following periods: no nociceptive stimuli (T15-T20), skin-muscle-peritoneal incisions (T30-T35), intestinal manipulation (T35-T40), peritoneal-muscle sutures (T40-T45), and skin sutures (T50-T55).

### 2.10 Hematological and stress hormone analyses

BGL was assessed using a glucose analyzer (NIPRO Stat Strip XP2, Nipro Corporation, Osaka, Japan). Hb concentrations were measured using an automated hematology analyzer (Celtac *α* MEK-6550, Nihon Kohden Corporation, Tokyo, Japan). Norepinephrine (NE; known as noradrenaline), epinephrine (EPI; known as adrenaline), and CSL were analyzed from samples stored at −80 °C by SRL, Inc. (Tokyo, Japan). Hematological and stress hormone analyses were performed on arterial blood samples collected at BL and at 15-min intervals (T15, T30, T45, and T60). The values at BL, T15, and T30 represent values before the nociceptive stimulus. T45 represents values during nociceptive stimulation, and T60 represents values after the nociceptive stimulus.

### 2.11 Statistical analysis

Statistical analyses were performed using JMP software (ver. 17.0, JMP Statistical Discovery LLC). The normality of continuous variables was assessed using the Shapiro–Wilk test. Since the RT variable showed a normal distribution, a two-way analysis of variance (ANOVA) was performed to analyze the effects of time, group, and their interaction. A generalized linear mixed model (GLMM) with a gamma distribution was employed to analyze differences between groups and time points, and interactions, for non-normally distributed variables (HR, MAP, SAP, DAP, pHa, PaCO_2_, EtCO_2_, SaO_2_, LF, HF, LF (n.u.), HF (n.u.), LF/HF, Hb, BGL, NE, EPI, and CSL). Dunnett’s test or Tukey’s HSD test was conducted for variables that demonstrated significant differences between time points and interactions. Likelihood ratio tests were performed to compare palpebral and corneal reflexes between groups. The recovery time of the swallowing reflex was analyzed using the Wilcoxon rank-sum test. The significance of differences was defined as *p* < 0.05.

## 3 Results

### 3.1 Effects of cardiorespiratory function and hematological parameters

Table 2 shows *p*-values between groups, time points, and their interactions analyzed by a two-way ANOVA or GLMM. Detailed information regarding cardiorespiratory and hematological parameters at each time point is presented in Table 3. There was no interaction between group and time point for RT (*p* = 0.8335). RT was significantly higher in the XBP group than the ISO group (*p* = 0.0040). Both groups showed the highest values at BL, which gradually decreased significantly as anesthesia progressed, reaching the lowest point at T60 (*p* < 0.0001). There was an interaction between group and time point for HR (*p* < 0.0001). In the XBP group, the highest HR values were observed at BL and significantly decreased throughout the anesthesia period (T10-T60; all *p* < 0.0001). Similarly, in the ISO group, HR was highest at BL and decreased significantly from T25 (*p* = 0.0029), with this effect persisting until T60 (*p* < 0.0001). On the other hand, no significant differences were observed between the two groups at the same time points. Significant interactions were observed between group and time point for MAP, SAP, and DAP (*p* < 0.0001, respectively). In the XBP group, MAP (T15-T55; *p* range 0.0092–0.0294) and DAP (T15-T55; *p* range 0.0192–0.0371) remained stable without significant differences from BL throughout anesthesia maintenance, maintaining significantly higher values compared to the ISO group. In the ISO group, MAP, SAP, and DAP were highest at BL and decreased significantly as anesthesia progressed (T10-T60; all *p* < 0.0001). There was no interaction between group and time point for pHa (*p* = 0.1294). There were no significant differences in pHa between the two groups (*p* = 0.2405). In both groups, pHa was lowest at BL and increased significantly from T30 (*p* = 0.0020) to T60 (*p* = 0.0008). There was no interaction between group and time point for PaCO₂ (*p* = 0.2632). In both groups, PaCO₂ was highest at BL and, except for T30, significantly decreased from T15 (*p* = 0.0113) to T60 (*p* = 0.0077) compared to BL. Additionally, the ISO group demonstrated significantly higher PaCO₂ values than the XBP group throughout anesthesia maintenance (*p* = 0.0003). There was no interaction between group and time point for SaO_2_ (*p* = 0.9988). In both groups, SaO₂ was significantly higher than BL at all time points (T15-T60; all *p* < 0.0001), and no significant differences were observed between groups (*p* = 0.9272). There were interactions between group and time point for Hb (*p* = 0.0162). The ISO group exhibited its highest values at baseline, followed by significant decreases during anesthesia maintenance (T15-T60; *p* range < 0.0001–0.0002). There were no interactions between group and time point for BGL (*p* = 0.1723). BGL was lowest at baseline in both groups and increased significantly during anesthesia maintenance (T15 through T60, *p* < 0.0001, respectively). EPI levels were significantly lower than BL at all time points (T15-T60; all *p* < 0.0001) in both groups. However, in the ISO group, there was a significant increase T60 compared to T30(*p* < 0.0170).

**Table 2:** Effects of group and time point on cardiorespiratory function, hematological, and heart rate variability parameters; probability values were analyzed using a two-way ANOVA or GLMM.

Parameter	Group	Time point	Interaction (Group × Time point)	Statistical analysis
RT	0.0040	<0.0001	0.8335	ANOVA
HR	0.3793	<0.0001	<0.0001	GLMM
MAP	0.0005	<0.0001	<0.0001	GLMM
SAP	0.0120	<0.0001	<0.0001	GLMM
DAP	0.0003	<0.0001	<0.0001	GLMM
pHa	0.2405	0.0009	0.1294	GLMM
PaCO_2_	0.0003	0.0060	0.2632	GLMM
EtCO_2_	0.3128	0.5282	0.1671	GLMM
SaO_2_	0.9272	<0.0001	0.9988	GLMM
Total power	0.0076	0.0004	0.0003	GLMM
LF	0.0005	<0.0001	0.0212	GLMM
HF	0.0091	0.0012	0.0002	GLMM
LF (n.u.)	0.1677	0.0011	0.1250	GLMM
HF (n.u.)	0.1718	0.0049	0.7145	GLMM
LF/HF	0.1588	0.0005	0.1079	GLMM
Hb	0.1885	<0.0001	0.0162	GLMM
BGL	0.6822	<0.0001	0.1723	GLMM
NE	0.9042	<0.0001	0.3864	GLMM
EPI	0.3469	<0.0001	0.0432	GLMM
CSL	0.0178	<0.0001	0.0009	GLMM

RT rectal temperature, HR heart rate bpm beats per minute, MAP mean arterial blood pressure, SAP systolic arterial blood pressure, DAP diastolic arterial blood pressure, pHa pH of arterial blood, PaCO_2_ partial pressure of arterial carbon dioxide, EtCO_2_ end-tidal carbon dioxide pressure, SaO_2_ arterial oxygen saturation, Hb hemoglobin, BGL blood glucose level, LF (ms^2^) low-frequency (0.04–0.10 Hz), HF (ms^2^) high-frequency (0.10–0.80 Hz), LF (n.u.) normalized LF = LF/ (Total power−very-low-frequency component (VLF)) × 100), HF (n.u.) normalized HF = HF/ (Total power−VLF) × 100), LF/HF LF/HF ratio, NE norepinephrine, EPI epinephrine, and CSL cortisol.

**Table 3:** Cardiorespiratory function and hematological parameters in 14 calves before anesthesia (baseline) and during 60 min of the continuous infusion of xylazine-butorphanol-propofol (XBP) or inhalation anesthesia with isoflurane (ISO).

Parameter	Group	Baseline	Time point										
			T10	T15	T20	T25	T30	T35	T40	T45	T50	T55	T60
RT (°C)^*^	XBP	39.0 ± 0.4	38.7 ± 0.7	38.5 ± 0.6	38.3 ± 0.7^$^	38.3 ± 0.8^$^	38.2 ± 0.6^$^	38.0 ± 0.8^$^	38.0 ± 0.7^$^	37.9 ± 0.8^$^	37.8 ± 0.8^$^	37.8 ± 0.9^$^	37.6 ± 0.8^$^
	ISO	39.2 ± 0.5	38.6 ± 0.5	38.5 ± 0.5	38.4 ± 0.5^$^	38.1 ± 0.5^$^	37.9 ± 0.6^$^	37.7 ± 0.5^$^	37.6 ± 0.3^$^	37.4 ± 0.4^$^	37.4 ± 0.4^$^	37.2 ± 0.4^$^	37.0 ± 0.5^$^
HR (bpm)	XBP	73 [54–107]	55 [36–77]^$^	55 [36–71]^$^	57 [36–73]^$^	58 [37–67]^$^	58 [37–66]^$^	58 [37–66]^$^	58 [38–66]^$^	61 [39–66]^$^	44 [38–66]^$^	55 [38–66]^$^	54 [38–65]^$^
	ISO	64 [50–113]	65 [55–79]	65 [53–74]	62 [50–72]	61 [48–70]^$^	59 [47–68]^$^	58 [46–69]^$^	57 [45–69]^$^	56 [44–69]^$^	55 [42–68]^$^	54 [41–68]^$^	53 [41–67]^$^
MAP (mmHg)	XBP	102 [95–124]	114 [64–136]	104 [64–139]^†^	111 [70–141]^†^	114 [62–142]^†^	114 [65–146]^†^	111 [71–150]^†^	109 [64–153]^†^	106 [63–158]^†^	105 [67–152]^†^	107 [70–149]^†^	107 [67–148]
	ISO	112 [96–160]	68 [57–102]^$^	63 [53–77]^$^	61 [49–70]^$^	60 [48–72]^$^	62 [45–73]^$^	63 [44–75]^$^	59 [47–67]^$^	56 [54–78]^$^	66 [48–84]^$^	74 [46–84]^$^	72 [46–85]^$^
SAP (mmHg)	XBP	128 [117–152]	134 [85–154]	136 [78–159]	135 [83–158]	133 [80–161]	130 [80–156]	130 [86–165]	124 [72–171]	120 [78–175]	120 [78–177]	121 [80–172]	121 [80–172]
	ISO	136 [126–176]	100 [82–123]^$^	95 [83–107]^$^	94 [79–105]^$^	101 [73–104]^$^	97 [73–103]^$^	94 [77–101]^$^	91 [66–102]^$^	87 [73–106]^$^	95 [67–109]^$^	95 [72–109]^$^	101 [74–108]^$^
DAP (mmHg)	XBP	83 [65–114]	98 [46–128]	91 [52–131]^†^	91 [59–133]^†^	91 [46–134]^†^	93 [53–138]^†^	89 [65–140]^†^	81 [56–140]^†^	91 [54–144]^†^	87 [58–136]^†^	90 [61–134]^†^	84 [56–133]
	ISO	88 [66–150]	55 [40–84]^$^	45 [34–61]^$^	48 [35–58]^$^	49 [32–59]^$^	48 [30–64]^$^	50 [29–56]^$^	46 [35–58]^$^	44 [41–55]^$^	57 [29–62]^$^	60 [28–66]^$^	60 [28–72]^$^
pHa	XBP	7.38 [7.34–7.42]	NR	7.39 [7.11–7.50]	NR	NR	7.42 [7.36–7.52]^$^	NR	NR	7.42 [7.36–7.48]^$^	NR	NR	7.43 [7.37–7.50]^$^
	ISO	7.38 [7.29–7.42]	NR	7.44 [7.42–7.50]	NR	NR	7.44 [7.39–7.51]^$^	NR	NR	7.44 [7.42–7.50]^$^	NR	NR	7.44 [7.42–7.50]^$^
PaCO_2_ (mmHg)^*^	XBP	45.0 [41.0–49.4]	NR	30.6 [19.0–40.3]^$^	NR	NR	33.0 [25.6–42.9]	NR	NR	31.6 [20.8–42.1]^$^	NR	NR	35.3 [22.4–37.0]^$^
	ISO	44.8 [39.8–118.4]	NR	47.6 [38.1–56.7]^$^	NR	NR	45.7 [36.4–94.5]	NR	NR	44.8 [41.5–53.5]^$^	NR	NR	44.1 [39.0–49.3]^$^
EtCO_2_ (mmHg)	XBP	NR	NR	38 [34–41]	NR	NR	36 [34–38]	NR	NR	36 [35–39]	NR	NR	35 [35–41]
	ISO	NR	NR	35 [34–39]	NR	NR	36 [35–37]	NR	NR	35 [34–39]	NR	NR	37 [35–38]
SaO_2_ (%)	XBP	95.6 [92.5–97.7]	NR	99.8 [99.4–100.0]^$^	NR	NR	99.9 [99.5–99.9]^$^	NR	NR	99.9 [99.3–99.9]^$^	NR	NR	99.9 [99.6–99.9]^$^
	ISO	96.4 [89.8–97.8]	NR	99.8 [99.6–99.9]^$^	NR	NR	99.8 [99.0–99.9]^$^	NR	NR	99.9 [99.4–99.9]^$^	NR	NR	99.8 [99.6–99.9]^$^
Hb (g/dL)	XBP	10.0 [7.30–10.80]	NR	9.50 [7.40–10.10]	NR	NR	9.50 [7.50–10.50]	NR	NR	9.50 [7.50–10.20]	NR	NR	9.60 [7.50–10.60]
	ISO	8.90 [8.00–11.70]	NR	8.10 [7.40–10.40]^$^	NR	NR	8.20 [7.00–10.10]^$^	NR	NR	7.90 [7.00–10.10]^$^	NR	NR	8.10 [7.10–10.30]^$^
BGL (mg/dL)	XBP	65 [39–77]	NR	89 [75–160]^$^	NR	NR	100 [80–224]^$^	NR	NR	127 [103–238]^$^	NR	NR	126 [109–277]^$^
	ISO	53 [43–76]	NR	107 [94–180]^$^	NR	NR	111 [129–219]^$^	NR	NR	140 [125–250]^$^	NR	NR	153 [138–269]^$^

Data are expressed as the mean ± standard deviation or median [minimum-maximum] of 7 calves in each group. The normality of continuous variables was assessed using the Shapiro–Wilk test. Regarding normally distributed variables (RT), a two-way ANOVA of variance was employed to analyze differences between groups and time points, as well as their interactions. Concerning non-normally distributed variables (HR, MAP, SAP, DAP, pHa, PaCO_2_, EtCO_2_, SaO_2_, Hb, and BGL), a GLMM with a gamma distribution was employed to analyze differences between groups and time points, and their interactions. As a post hoc test, RT, HR, MAP, SAP, DAP, and Hb at baseline and other time points in each group as well as at the same time points between the two groups were analyzed by Tukey’s HSD test. pHa, SaO_2_, PaCO_2_, and BGL between baseline and other time points were analyzed using Dunnett’s test. Asterisks (*) indicate significant differences (*p* < 0.05) between the XBP and ISO groups. Dagger (†) indicates a significant difference (*p* < 0.05) between the XBP and ISO groups at the same time point. Dollar ($) indicates a significant difference (*p* < 0.05) between baseline and the indicated time points in each group. RT rectal temperature, HR heart rate bpm beats per minute, MAP mean arterial blood pressure, SAP systolic arterial blood pressure, DAP diastolic arterial blood pressure, pHa pH of arterial blood, PaCO_2_ partial pressure of arterial carbon dioxide, EtCO_2_ end-tidal carbon dioxide pressure, SaO_2_ arterial oxygen saturation, Hb hemoglobin, BGL blood glucose level, NR no reported data, LF (ms^2^) low-frequency (0.04–0.10 Hz), HF (ms^2^) high-frequency (0.10–0.80 Hz), LF (n.u.) normalized LF = LF/(Total power−very-low-frequency component (VLF)) × 100), HF (n.u.) normalized HF = HF/ (Total power−VLF) × 100), and LF/HF LF/HF ratio.

### 3.2 Depth of anesthesia and quality of recovery

**Figure 1:**
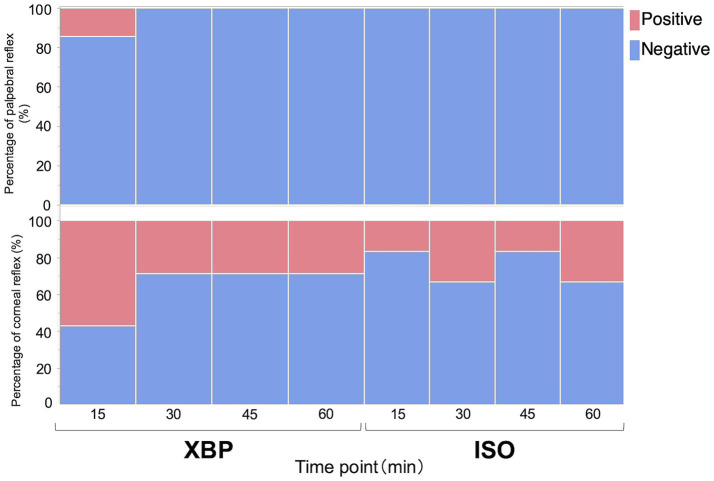
Evaluation of palpebral and corneal reflexes with xylazine-butorphanol-propofol (XBP) and isoflurane (ISO) anesthesia in calves. Data are expressed as a percentage of 7 calves in the XBP group and 6 calves in the ISO group. Likelihood ratio tests were performed for palpebral and corneal reflexes to compare between the groups. No significant differences were observed between the two groups.

As shown in Figure 1, no significant differences were observed in the palpebral (*p* = 0.9980) or corneal reflex (*p* = 0.3980) during anesthesia maintenance between the two groups. In addition, defensive responses against surgical stimuli were not observed during the laparotomy procedure. As shown in Table 4, the swallowing reflex time was significantly longer in the XBP group than in the ISO group (*p* = 0.0152).

**Table 4:** Evaluation of recovery from anesthesia.

Variable	XBP	ISO
Swallowing reflex recovery time (min)	38.0 [16.9–91.2]^*^	15.6 [11.5–26.0]

Data are expressed as the median [minimum-maximum] of 7 calves in each group. The results of the Shapiro–Wilk test showed that the swallowing reflex recovery time was not normally distributed. Therefore, the Wilcoxon rank-sum test was used to analyze group differences. Asterisks (*) indicate significant differences (*p* < 0.05) between the two groups.

### 3.3 HRV analysis

Detailed information regarding HRV parameters at each time point is presented in Table 5. There were interactions between group and time point for Total power (*p* = 0.0003). Total power was significantly higher in the XBP group than in the ISO group for no nociceptive stimulus (*p* = 0.0069) and skin and muscle incisions (*p* = 0.0094). In the XBP group, total power showed the highest values at the no nociceptive stimulus point and demonstrated significantly lower values during muscle suture (*p* = 0.0007) and skin suture (*p* = 0.0002) compared to the no nociceptive stimulus point. There were interactions between group and time point for LF (*p* = 0.0212). LF was significantly higher in the XBP group than in the ISO group at all time points (no nociceptive stimulus, *p* = 0.0002; skin sutures, *p* = 0.0267). There were interactions between group and time point for HF (*p* = 0.0002). HF was significantly higher in the XBP group than in the ISO group for no nociceptive stimulus (*p* = 0.0077) and skin and muscle incisions (*p* = 0.0153). In the XBP group, HF showed the highest values at the periods of no nociceptive stimulus and demonstrated significantly lower values during the periods of muscle suture (*p* = 0.0003) and skin suture (*p* = 0.0002) compared to the periods of no nociceptive stimulus. No interactions between group and time were observed for LF (n.u.) (*p* = 0.1250), HF (n.u.) (*p* = 0.7145), and LF/HF (*p* = 0.1079). LF (n.u.) (*p* = 0.0039) and LF/HF (*p* = 0.0020) showed significant increases compared to periods of no nociceptive stimulation, reaching maximum values. In contrast, HF (n.u.) showed statistically significant decreases compared to periods of no nociceptive stimulation (*p* = 0.0097), reaching minimum values.

**Table 5:** Heart rate variability parameters in 13 calves during 60 min of the continuous infusion of xylazine-butorphanol-propofol (XBP) or inhalation anesthesia with isoflurane (ISO).

Parameter	Group	No nociceptive stimulus	Time point of the nociceptive stimulus procedure			
			Skin and muscle incisions	Intestinal manipulations	Muscle sutures	Skin sutures
		(T15-T20)	(T30-T35)	(T35-T40)	(T40-T45)	(T50-T55)
Total power (ms^2^)	XBP	1247.40 [5.95–24055.24]^†^	804.72 [10.89–16559.14]^†^	524.84 [4.56–7411.54]	189.72 [1.74–1517.23]^$^	172.37 [4.24–1211.81]^$^
	ISO	2.92 [1.40–53.02]	5.79 [1.87–83.32]	9.58 [2.05–55.64]	6.56 [1.93–125.53]	5.78 [1.15–132.22]
LF (ms^2^)	XBP	22.05 [1.26–683.98]^†^	53.93 [1.47–438.25]^†^	27.95 [4.25–459.46]^†^	28.83 [0.04–121.66]^†^	4.84 [0.03–56.60]^†$^
	ISO	0.16 [0.01–0.98]	0.24 [0.02–1.14]	0.71 [0.10–7.77]^$^	0.13 [0.03–0.88]	0.13 [0.03–0.24]
HF (ms^2^)	XBP	1211.51 [3.33–23073.92]^†^	729.53 [6.68–15922.69]^†^	448.52 [7.27–6753.19]	154.35 [1.41–1463.72]^$^	166.07 [4.20–1177.77]^$^
	ISO	2.81 [1.10–52.81]	3.86 [1.63–82.80]	8.11 [1.77–54.75]	6.36 [1.82–125.31]	5.74 [1.02–132.04]
LF (n.u.)	XBP	4.22 [1.70–27.4]	11.53 [2.68–17.05]	19.91 [2.18–41.65]^$^	13.45 [0.77–27.16]	3.31 [0.71–18.01]
	ISO	4.96 [0.03–15.30]	5.13 [0.10–22.79]	9.89 [0.83–42.69]^$^	3.39 [0.14–6.22]	2.96 [0.11–7.92]
HF (n.u.)	XBP	95.78 [72.60–98.30]	88.47 [82.95–97.32]	80.09 [58.35–97.82]^$^	86.55 [72.84–99.23]	96.69 [81.99–99.29]
	ISO	95.04 [84.70–99.97]	94.87 [77.21–99.90]	90.11 [57.31–99.17]^$^	96.61 [93.78–99.86]	97.04 [92.08–99.89]
LF/HF	XBP	0.04 [0.02–0.38]	0.13 [0.03–0.21]	0.27 [0.02–0.71]^$^	0.16 [<0.01–0.37]	0.03 [<0.01–0.22]
	ISO	0.05 [<0.01–0.18]	0.05[<0.01–0.30]	0.11 [<0.01–0.75]^$^	0.04 [<0.01–0.07]	0.03 [<0.01–0.09]

Data are expressed as the median [minimum-maximum] of 6 calves in the XBP group and 7 calves in the ISO group. The normality of continuous variables was assessed using the Shapiro–Wilk test. Regarding non-normally distributed variables (Total power, LF, HF, LF (n.u.), HF (n.u.), and LF/HF), a GLMM with a gamma distribution was employed to analyze differences between groups and time points, and their interactions. As a post hoc test, Total power, LF, and HF between baseline and other time points in each group as well as at the same time points between the two groups were analyzed by Tukey’s HSD test. LH (n.u.), HF (n.u.), and LF/HF at baseline and other time points were analyzed by Dunnett’s test. Dagger (†) indicates a significant difference (*p* < 0.05) between the XBP and ISO groups at the same time point. Dollar ($) indicates a significant difference (*p* < 0.05) between no nociceptive stimulus and the time point of the nociceptive stimulus procedure in each group. LF (ms^2^) low-frequency (0.04–0.10 Hz), HF (ms^2^) high-frequency (0.10–0.80 Hz), LF (n.u.) normalized LF = LF/ (Total power−very-low-frequency component (VLF)) × 100), HF (n.u.)) × 100), and LF/HF LF/HF ratio (5).

### 3.4 Stress hormone parameter analysis

**Figure 2:**
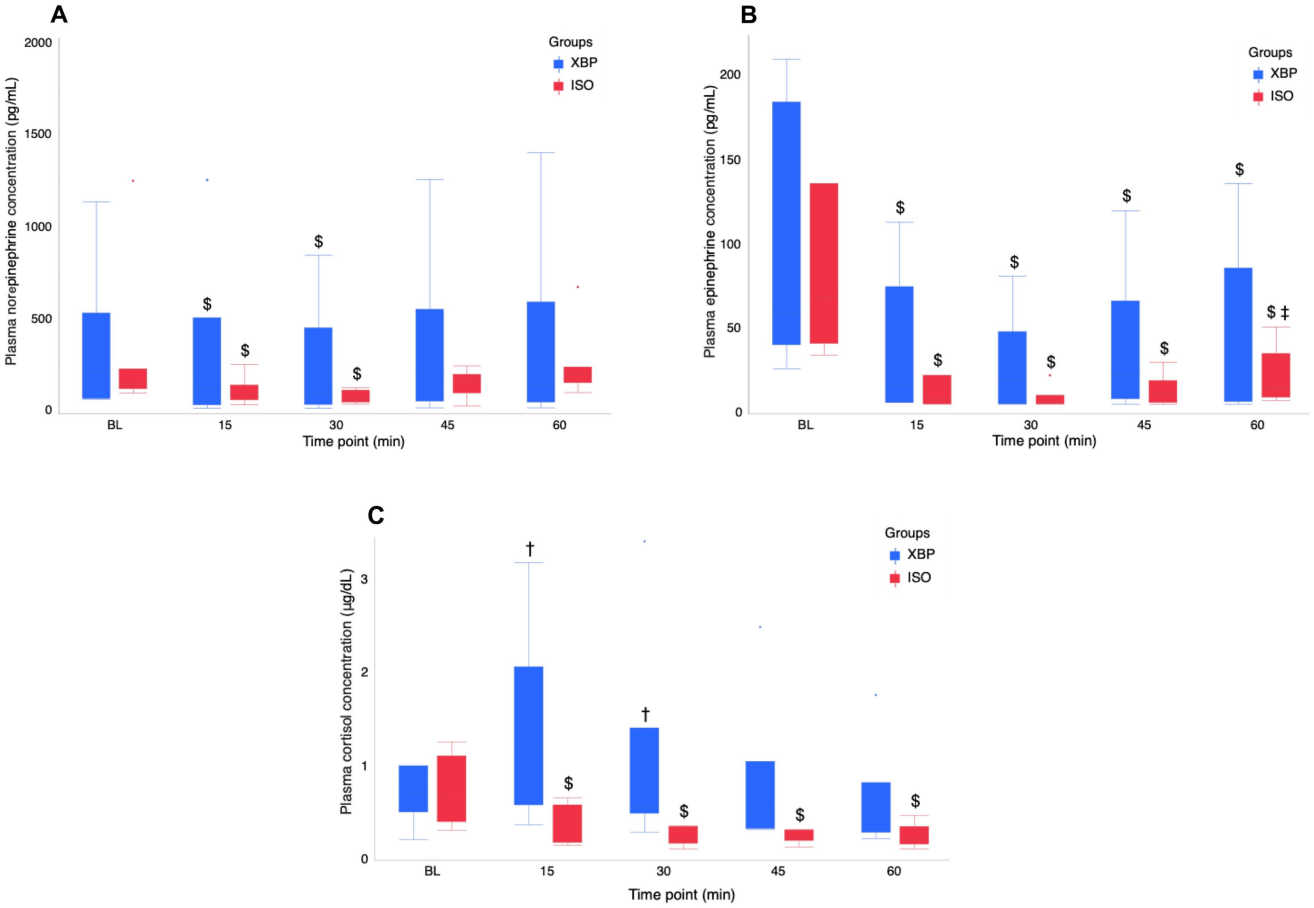
Temporal changes in stress hormone (NE **(A)**, EPI **(B)**, and CSL **(C)**) during the maintenance of anesthesia with the xylazine-butorphanol-propofol combination (XBP) versus isoflurane (ISO) in calves. Stress hormones analyses were performed on arterial blood samples collected at BL and at 15-min intervals (T15, T30, T45, and T60). Data are expressed as the median [minimum-maximum] of 6 calves in the XBP group and 7 calves in the ISO group. NE and EPI values < 5 pg./dL (detection limit) were set to 5 pg./dL. The normality of continuous variables was assessed using the Shapiro–Wilk test. Regarding non-normally distributed variables (NE, EPI, and CSL), a GLMM with a gamma distribution was employed to analyze differences between groups and time points, and their interactions. As a *post hoc* test, NE at BL and other time points was analyzed by Dunnett’s test. EPI at BL and other time points was analyzed by Tukey’s HSD test. The values at BL, T15, and T30 represent pre-nociceptive stimulation values. T45 represents values during nociceptive stimulation, and T60 represents values post-nociceptive stimulation. Dagger (†) indicates a significant difference (*p* < 0.05) between the XBP and ISO groups at the same time point. Dollar ($) indicates a significant difference (*p* < 0.05) between baseline and time points in each group. Double dagger (‡) indicates a significant difference (*p* < 0.05) between T30 and T60 (post-nociceptive stimulus procedure). BL, baseline; NE, norepinephrine; EPI, Epinephrine; CSL, Cortisol.

Detailed information regarding the stress hormone parameter at each time point is presented in Figure 2 and Supplementary Table 1. Significant interactions between group and time point were observed for EPI (*p* = 0.0432) and CSL (*p* = 0.0009), but not for NE (*p* = 0.3864). NE levels significantly decreased in both groups before nociceptive stimulus (T15, *p* = 0.0005; T30, *p* < 0.0001) compared to BL. However, during nociceptive stimulus (T45, *p* = 0.0626) and after nociceptive stimulus (T60, *p* = 0.6421), values increased and no longer showed significant differences from BL. EPI levels were significantly lower than BL at all time points (T15-T60; all *p* < 0.0001) in both groups. However, in the ISO group, there was a significant increase T60 compared to T30(*p* < 0.0170). CSL in the XBP group showed the highest values at T15 and was significantly higher than the ISO group before nociceptive stimulus (T15, *p* = 0.0356; T30, *p* = 0.0403). However, after T45 (*p* = 0.1642), no significant differences were observed between the groups. The ISO group showed the highest BL values, with significantly lower values during anesthesia maintenance compared to BL (T15-T60; *p* range < 0.0001–0.0444).

## 4 Discussion

In the present study, ISO inhalation anesthesia, which has been widely studied as a standard method for general anesthesia in cattle, was used as the control group. It has been reported that ISO inhalation anesthesia cause hypotension and hypothermia in proportion to the duration of anesthesia (20). Therefore, we evaluated the clinical significance of the XBP group by comparing it with the ISO group.

Although XBP anesthesia decreases RT during anesthesia, it maintains temperature better than ISO anesthesia. Previous studies in rats (21) and humans (22) reported that ISO maintenance anesthesia reduced body temperature due to peripheral vasodilation, which causes heat loss through the skin, and suppression of the thermoregulatory center in the hypothalamus. The mechanism by which body temperature decreases during general anesthesia involves increased skin blood flow due to peripheral vasodilation. Warm blood is redistributed from the core to the surface of the skin. An increased skin surface temperature leads to heat loss, which, in turn, decreases body temperature (22). Furthermore, severe hypothermia in calves is defined as a body temperature below 37.0 °C (23) and hypothermia causes delayed postoperative recovery and surgical site infections (21). In the present study, most calves in the XBP group maintained body temperatures above 37.0 °C throughout anesthesia maintenance. Therefore, XBP anesthesia may represent a safer anesthetic procedure compared to ISO anesthesia due to a lower risk of hypothermia during surgery, at least within in 60-min anesthesia period.

Although the calives exhibited lower HR during anesthesia than BL, there was no differences between the XBP and ISO groups. In calves, bradycardia is defined as a HR below 80 beats per minute (bpm) (23). In the present study, most calves in both groups exhibited HR below 80 bpm during the anesthesia maintenance phase. On the other hand, BLs for isoflurane inhalation anesthesia in calves have been reported as 65 ± 12 bpm (24). General anesthesia typically induces bradycardia, which can cause a decrease in arterial blood pressure (20). Therefore, further studies are required to develop measures to address bradycardia in calves during general anesthesia calves.

In the present study, MAP in the XBP group was significantly higher than in the ISO group. Arterial blood pressure is measured by the product of cardiac output and systemic vascular resistance. Cardiac output, in turn, is calculated as the product of stroke volume and HR. (25) Typically, a decrease in HR would result in a concomitant decrease in arterial pressure. However, in the XBP group, MAP during anesthesia maintenance was comparable to BL and significantly higher than in the ISO group. This suggests that the bradycardia induced by propofol and xylazine was effectively compensated. In addition, the XBP group exhibited considerably elevated arterial blood pressure throughout the duration of the anesthesia maintenance phase. This was attributed to the vasoconstrictive effects of xylazine (1) adequately counterbalancing the vasodilatory effects of propofol and butorphanol. In addition, many calves in the ISO group exhibited lower MAP than 70 mmHg, defined as hypotension (20), during anesthesia maintenance. In contrast, the XBP group maintained BL levels throughout the anesthesia maintenance period. Therefore, these results suggest that XBP anesthesia provides superior hemodynamic stability compared to ISO anesthesia.

In the present study, respiratory management was conducted to maintain EtCO_2_ at 35–40 mmHg during anesthesia in both groups. Consequently, no significant differences in EtCO_2_ were observed between groups or time points. However, PaCO_2_ was significantly higher in the ISO group than in the XBP group throughout anesthesia. The condition where PaCO_2_ remains elevated despite constant EtCO_2_ may be attributed to an increased ventilation-perfusion mismatch (V/Q mismatch). ISO reduces the systemic vascular resistance in a dose-dependent manner through its vasodilatory effects, leading to decreased MAP during anesthesia maintenance (26). Furthermore, positive pressure ventilation under ISO anesthesia has been reported to increase physiological dead space (27). These factors wer considered to contribute to a ventilation-perfusion mismatch, resulting in an effective hypoventilation state and consequently elevated PaCO_2_ in the present study. A previous study has shown that while maintaining XBP anesthesia under spontaneous breathing preserved adequate oxygenation, it resulted in hypercapnia and acidemia (1). In the XBP group, mechanical ventilation effectively prevented hypercapnia through enhanced elimination of carbon dioxide from the body. In addition, SaO₂ remained within the normal range for cattle throughout anesthesia maintenance, indicating adequate oxygenation. These results suggested that XBP anesthesia achieved superior ventilation efficiency to ISO anesthesia during mechanical ventilation.

The present results showed that XBP anesthesia administered at a rate of 6 mL/kg/h produced an anesthetic depth equivalent to ISO anesthesia (EtIso: 1.3 ± 0.1%) in calves. The reported 1MAC for cattle ranges between 1.27 and 1.47% (26, 28). Palpebral and corneal reflexes are standard indicators for evaluating the depth of anesthesia in cattle (1, 25). In the present study, no significant differences were observed in the presence or absence of palpebral and corneal reflexes between the two groups. Furthermore, neither group exhibited notable aversive responses to nociceptive stimuli or behaviors that may impede surgical progress. Recovery from anesthesia was slower with XBP anesthesia than with ISO anesthesia. In the present study, quality of recovery from anesthesia was assessed only by the swallowing reflex as the recovery time. After the swallowing reflex was restored, all calves were extubated and confirmed to stand after being intravenously administered atipamezole. Isoflurane is primarily eliminated through ventilation (26), resulting in a rapid decrease in blood concentration when administration is discontinued. This enables relatively prompt recovery from anesthesia. In contrast, TIVA may lead to drug accumulation in the patient’s body during prolonged continuous administration, potentially causing delayed drug elimination and prolonged recovery (26). Consequently, XBP may result in more prolonged anesthetic recovery compared to ISO due to residual drug effects.

In the present study, the HRV analysis revealed that calves in the XBP group exhibited significantly higher values than the ISO group during periods without nociceptive stimulus and during skin and muscle incisions. This result indicates that ANS activity was suppressed more gradually in the XBP group compared to the ISO group. Total power in HRV represents the overall activity of the ANS, with higher values signifying greater adaptability to environmental changes (29). The suppression of autonomic nervous activity was clearly observed in the ISO groups from the early phases of anesthesia maintenance. The ISO administration reduces total power during anesthesia (30, 31). In the present study, the ISO group demonstrated reduced autonomic responses to hypothermia (32) and hypotension (20) that can occur during anesthesia.

In the present study, while no significant difference in HF was observed during intestinal manipulation compared to periods without nociceptive stimulation, HF (n.u.) significantly decreased only during intestinal manipulation among all nociceptive stimuli examined. The decreased HF components in HRV serve as indicators of pain or unpleasant stimuli (33). This result indicates a relative reduction in PNS activity specifically during intestinal manipulation. The LF/HF ratio significantly increased following intestinal manipulation in comparison to the absence of nociceptive stimulation. In cattle study, the LF/HF is considered to represent the balance between SNS and PNS (15), and an increase in the LF/HF ratio suggests sympathetic predominance. Therefore, our findings suggest that intestinal manipulation triggered a nociceptive stress response in calves. However, in human studies, the LF/HF ratio alone cannot adequately assess ANS balance (34). Therefore, we conducted additional assessments using stress hormone indicators to provide a more comprehensive evaluation of ANS responses.

In the analysis of stress hormone, plasma NE levels in both groups decreased significantly after the initiation of anesthesia maintenance but recovered to BL levels following nociceptive stimuli. Additionally, plasma EPI concentrations during anesthesia maintenance were significantly lower than BL values in both groups. However, only the ISO group exhibited a significant increase in plasma EPI before and after nociceptive stimuli, indicating an SNS response to nociceptive stimuli. In the results of the present study, the XBP group demonstrated superior anti-nociceptive effects compared to the ISO group.

The XBP group had significantly higher plasma CSL values than the ISO group at T15 and T30. However, values in the XBP group decreased from T45 to T60. In contrast, plasma CSL values in the ISO group exhibited a persistent and substantial decrease relative to BL values during the maintenance phase of anesthesia. Volatile inhalation anesthetics have been demonstrated to suppress hypothalamic activation in response to stress, thereby leading to reduced cortisol secretion (8). Consequently, the ISO group demonstrated suppressed hypothalamic activity from the early stages of anesthesia. Conversely, plasma cortisol levels in calves have been reported to increase due to handling (35). Therefore, the XBP group maintained autonomic nervous system responsiveness during the initial stages of anesthesia maintenance, suggesting that stress responses to handling occurred. Further research on general anesthesia protocols with anti-nociceptive effects in calves will contribute to the implementation of safe and effective surgical procedures, addressing a critical challenge in veterinary clinical practice.

### 4.1 Limitation

In the present study has several limitations. First, it was conducted as a single-center, open-label investigation with a sample size of 14 subjects. While this size was sufficient to detect major differences, it may have limited the ability to detect subtle intergroup variations. Then, the inclusion of calves of varying breeds, sexes, ages, and weights may have introduced physiological variability, which could have affected the responses to anesthesia. In addition, regarding the health status of the calves, three in the XBP group were classified as ASA Grade II, compared to only one in the ISO group. This classification stemmed from minor localized issues that distinguished them from calves in optimal health. Since these animals maintained good systemic health, we concluded that these localized clinical signs had negligible impact on the results of the present study. Further research with additional clinical cases are required to investigate the effects of the body condition on the efficacy and risk of general anesthesia in calves.

## 5 Conclusion

The XBP group maintained higher body temperature and blood pressure during anesthesia maintenance compared to the ISO group and demonstrated better ventilation efficiency. Both groups showed sympathetic activation in response to visceral pain; however, the ISO group exhibited a more pronounced elevation of EPI in response to nociceptive stimulation. This finding suggests that the XBP protocol offers superior anti-nociceptive effects. These results support that TIVA using XBP represents a viable and potentially advantageous alternative to ISO inhalation anesthesia in calves.

## Data Availability

The original contributions presented in the study are included in the article/Supplementary material, further inquiries can be directed to the corresponding author.
